# Sampling migrants in six European countries: how to develop a comparative design?

**DOI:** 10.1186/s40878-018-0099-x

**Published:** 2018-10-29

**Authors:** Hans-Jürgen Andreß, Romana Careja

**Affiliations:** 1 Universität zu Köln, Cologne, Germany; 2 Syddansk Universitet, Odense, Denmark

**Keywords:** immigration, immigrants, sampling frames, population register, network sampling

## Abstract

This article discusses the possibilities and constraints of designing an identical or at least comparable sampling strategy across different European countries. It is based on expert reviews from six European Union member states that discuss the possibilities of sampling migrants in their respective countries. The country sample includes two countries from Northern Europe (Sweden, Denmark), two from Continental Europe (Germany, The Netherlands), and two from Southern Europe (Spain, Italy). After a discussion of various definitions of the target population and an overview of existing strategies to sample them, it is investigated which of them can be used in the six countries analyzed in the expert reviews. The focus is on probability samples and the use of population registers, while other sampling strategies are only briefly touched upon. The analysis shows that even with only six European countries an identical register-based sampling design is difficult. The authors propose that, by focusing on sampling immigrants in cities, researchers can better implement sampling strategies which result in comparable samples.

## Introduction

The increased influx of migrants into European countries presents a major challenge to official statistical bodies, survey researchers, and academics. The need for more comprehensive and reliable data has been acknowledged both by the European Union and by international statistical bodies such as the United Nations, the OECD, and the World Bank. Prominent examples are the EU regulation (EC) 862/2007 on community statistics on migration and international protection, the Declaration of Zaragoza (2010) stressing the need to have common indicators on migrants, or the Task Force on Improving Migration and Migrant Data Using Surveys and Other Data (also referred to as the ‘Suitland Working Group’) as well as the recent initiative *International Forum* on *Migration Statistics*, organized under the aegis of OECD, IOM and UNDESA. In response, and in parallel to these initiatives, many European countries have made efforts to improve their national statistical infrastructure to better account for immigrant minorities (for the advancements at Eurostat see Kraler, Reichel, & Entzinger, 2015, p. 44–49).

In this text we use the term “immigrant minorities” to designate ethnic minority groups who originate from migratory processes. The term has been around for long time and encompasses both recently arrived immigrants and individuals settled in the territory of the host countries, as well as their offspring. Another reason why we have opted to use this term is that, as this article and as the other three articles in this special issue show, countries differ in their official terminology. For example, Sweden does not officially use the terms “immigrants” or “descendants”, while some countries do (for example Denmark) and others use terms such as “ethnic minorities” in addition to “immigrants” (UK), “allochthon” (i.e., non-autochthonous) (The Netherlands), or “migration background” (Germany). The term “immigrant minorities” also allows a distinction from “national minorities”, who are ethnic groups who have lived in these European countries for centuries (for example Roma and Hungarians in Romania, Danes and Sorbs in Germany, Sami in the Nordic countries, Bascs in Spain and so forth). For texts using the term, see De Vroome, Hooghe, and Marien (2013), Dowley and Silver (2011), Geedes (1995), Hainmueller and Hangartner (2012), Schaeffer, Höhne, and Teney (2016), Schmitter (1983), Sniderman, Hagendoorn, and Prior (2004).

There is an undeniable and welcome increase in data capturing basic socio-demographic and socio-economic information about immigrant minorities. Cross-national survey projects such as the European Social Survey (ESS), the International Social Survey Programme (ISSP), the Eurobarometer surveys, the European Value Study (EVS) or the European Labour Force Survey (LFS) (which focuses only on the labor market situation) capture only a small proportion of immigrant minorities in each survey and survey waves have to be pooled to achieve sufficiently large sample sizes. Country-level surveys such as the German Socio-Economic Panel (SOEP) or the Longitudinal Internet Studies for the Social Sciences (LISS) in The Netherlands^1^ include larger samples, but are not comparable across countries as the sampling methodology and the questionnaires differ. Sometimes, country-level surveys focus only on certain regions within the country, such as the Osservatorio Regionale per L’integrazione e la Multietnicità in Lombardy, an Italian region. Moreover, all these surveys implement a module-system, with a set of basic variables collected regularly, and thematic variables collected at various time-intervals. Only recently several specialized survey programs on immigrant minorities have been launched, both national (e.g., the IAB-SOEP Migration Sample) and cross-national (see e.g. the NORFACE Research Programme on Migration or the EU research programs (FP7, HORIZON 2020) with several projects on migration).

There is a widespread consensus that for an in-depth understanding of integration processes and of effects of different reception contexts on these processes, detailed comparative data are necessary. Thus, two desired outcomes emerge: (i) data should be collected on immigrant minorities and relevant control groups selected in sufficient sample sizes and in an identical (comparable) way in all countries, and (ii) the data should allow for the formation of a comprehensive picture of their living situation focusing not only on their labor market position, but also on their incorporation in the destination countries’ social, cultural, and political spheres.

This article focuses on the first desideratum and discusses the possibilities and constraints of designing an identical or, at least, comparable sampling strategy across several European countries (for other methodological issues of migration surveys see Bonifazi, Okólski, Schoorl, & Simon, 2008; Font & Méndez, 2013; Vargas-Silva, 2012). One of the main problems of sampling immigrant minorities is the: “lack of readily available sampling frames from which to sample members of minority groups, including the second generation” (Groenewold & Lessard-Phillips, 2012, p. 39). Moreover, when it comes to international comparisons, the experience from many comparative research projects on migrants shows that it is difficult to develop and maintain an identical sampling strategy in all the countries being compared (see, e.g., Crossing Borders Making Europe (EUCROSS), Pioneers of Europe’s Integration‚ from below’ [PIONEUR] (Fernández, Rother, & Braun, 2006), or the Six Country Immigrant Integration Comparative Survey [SCIICS] (Ersanilli & Koopmans, 2013)).^2^

In 2015 a group of survey and migration experts from different European countries met to discuss the possibilities of sampling immigrant minorities in their respective countries. A selection of the evidence presented at this conference is published in three articles in this Special Issue (Careja & Bevelander, 2018; Salentin & Schmeets, 2017; Serrano Sanguilinda, Barbiano di Belgiojoso, González Ferrer, Rimoldi, & Blangiardo, 2017), covering six European destination countries: Denmark, Germany, Italy, The Netherlands, Spain, and Sweden. While these articles describe and discuss country-level possibilities and problems, this article takes the comparative view and asks whether, and how, a comparative sampling design could be developed for these countries.

Specifying a comparative sampling design starts with the problem of finding a common language defining the target population, identifying its members, and distinguishing different methods to sample them. “A frame of comparison: definitions, identification strategies, and sampling procedures” section, based on the expert discussions, abstracts from the country-specific definitions and practices presented in the expert reviews and provides a more general frame of comparison that encompasses all the national solutions, but also provides alternative options. Obviously, the six countries discussed in this Special Issue do not represent all European destination countries, but – as we argue in “The countries of our comparison” section – encompass a significant share of the population and immigrants in the European Union and, even more importantly, show enough variance with respect to their immigration history and statistical infrastructure to study the problems of comparative sampling designs. The information provided in the country-specific articles is summarized in “What is possible in Denmark, Germany, Italy, The Netherlands, Spain, and Sweden?” section with respect to four overall questions: (i) What are the basic concepts and definitions used in these countries? (ii) What information do the registers include to identify the population of interest? (iii) What population registers are available and how do they cover the population of interest? (iv) What other sampling registers and sampling methods exist? Based on this summary, the “Discussion and conclusion” section concludes with a discussion of whether and how a comparable sampling design would be feasible across these countries and which open questions remain.

## A frame of comparison: definitions, identification strategies, and sampling procedures

Any endeavor at sampling individuals with migration background must start with a definition of the population of interest. At first glance, defining who is immigrant seems evident. According to Regulation (EC) No 862/2007, Eurostat defines immigration as “the action by which a person establishes his or her usual residence in the territory of a Member State for a period that is, or is expected to be, of at least 12 months, having previously been usually resident in another Member State or a third country” (Art. 2, 1b). However, upon closer examination, this definition might not be sufficient because it focuses on first generation immigrants, specifically on those first-generation immigrants with a longer period of residence in the destination country. Thus, it needs to be complemented with other definitions (and the corresponding identification strategies) in order to capture second and third generation offspring, or, if the research question demands it, other categories of immigrants, such as short-term, temporary or circulatory.

However, one has to recognize an additional difficulty. Even if definitions such as the one mentioned above exist, they are not fully implemented when information about immigrants is reported. For example, Statistics Denmark uses the EU definition when reporting information on immigrants to Eurostat, but uses its own definitions domestically (Careja & Bevelander, 2018). Moreover, national discourses (and official statistics) acknowledge certain subgroups, but completely ignore others. Some countries, especially those with a long-standing immigration history from former colonies or with a strong focus on assimilation, would not use the term “immigrant” at all or use it only very selectively. For example, the UK uses both “ethnic minorities” and “immigrants”, where ethnic minorities are Indian, Pakistani, Black Caribbean, Black African, Bangladeshi and Chinese, while France acknowledges only different nationalities and denies completely the idea of “minorities”. To give another example: while many countries provide information on the offspring of the first-generation (“non-nationals”, “individuals with migration background”, “descendants”), Sweden reports them as Swedish-born (in the same category together with the native Swedes). Even the definition of “non-nationals” or “individuals with migration background” varies greatly between countries (see the country-specific articles in this Special Issue).

From the viewpoint of integration research, one may ask whether the terms “immigration” and “immigrants” fall short of the mark. As we will later demonstrate foreign-born individuals and those with a ‘different nationality’ are easy to track using what could be considered quite trivial data. However, when it comes to the question of integration, such simple definitions are only the start of the problem because markers of foreignness remain in local born generations. Most discrimination studies show that a foreign sounding name or a darker skin are enough to trigger differential treatment in labor markets no matter how integrated the person might otherwise be (for an overview see Kahanec & Zimmermann, 2011). US and UK statistics, for example, use terms such as race because it is obvious that a significant social difference remains many decades after immigration. Thus, integration research inevitably uses the term “ethnic minorities”, which – of course – has consequences for possible sampling strategies (for the methodological pitfalls of measuring race see Roth, 2017). Academic research has to leave official statistics behind and ask on the basis of the social, political and scholarly interest what is appropriate in a given situation. Minority identification may be such a thing (for some European desiderata see Simon, 2017). Indeed, many would doubt if the racial/ethnic identity of a person is something the state should register, yet if it is considered essential in order to understand the social structure, academics may consider it a necessary part of their toolkit. Immigrant minorities, the term used here, are only a subgroup of the more encompassing definition of ethnic minorities.

Given different research interests and different national discourses on immigration, it was important to define a common frame of reference (a “common language”) for the national experts participating in the conference. The frame aimed to provide a comprehensive definition of the target population that would include all national definitions as special cases. Furthermore, it aimed to provide a comprehensive list of identification.

**Fig. 1:**
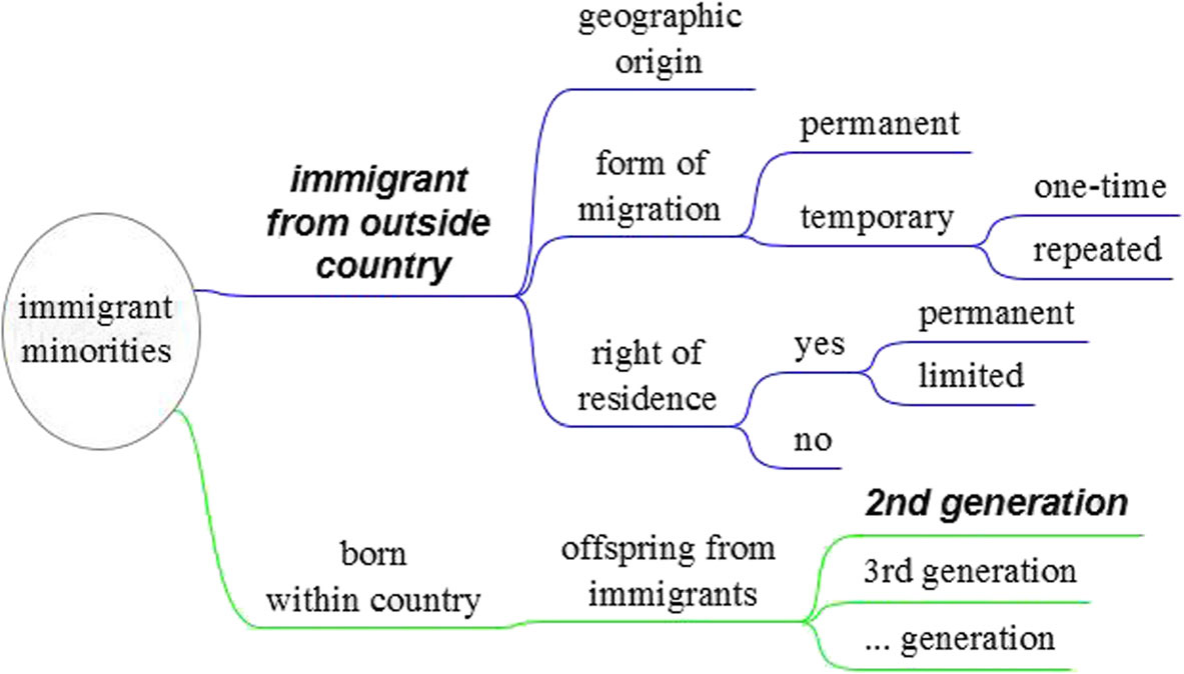
Immigrant minorities. *Notes*: Subgroups highlighted about which Eurostat provides statistical data

The experts agreed upon the term “immigrant minorities” as the most encompassing definition of the target population. Starting with immigrant minorities, one can distinguish between members of this population that immigrated from outside the country and members that were born within the country (see Fig. 1). Immigrant minorities having immigrated from outside the country (the first-generation immigrants) are at the core of official migration statistics and can be distinguished according to their geographic origin, their form of migration, and their right of residence. Immigrant minorities born within the country include the offspring of the first-generation immigrants living in the country.^3^

As already noted, integration into the host society is a life-long process, sometimes taking several generations. Hence, if one is interested in the process of integration, as most researchers are, ideally the definition of the target population ought to include a decision regarding the time during which migrants have arrived in the destination country or left the origin country. The choice of the time period depends on the mode of data collection: Should the process of integration be observed retrospectively or prospectively? Should only first-generation immigrants be sampled, or should also their children and grandchildren be included (if the process of integration takes several generations)? How far back into the past one has to go depends, among other things, on the history of migration in each country. However, it must be recognized that the decision on the different options is often a question of available resources.

Immigrant minorities are a very heterogeneous category. Figure 1 mentions some important characteristics that differentiate them: (i) their geographic origin, (ii) their right of residence, and (iii) the form of their immigration. In the optimal case, this information is available not only for first-generation immigrants themselves, but also for their offspring in order to identify those individuals that have been born in the country but have a migration background through their parents and grandparents.

When it comes to sampling immigrant minorities, the questions of how “visible” they are and how long they have been present in the country are of utmost importance. Since sampling is more difficult for immigrants without residence rights (so-called “irregular” or “unregistered” migrants) or those with only temporary migration intentions (temporary or circulatory migration), we mainly discuss sampling issues with respect to those immigrant minorities that have residence rights and intend to remain in the destination country permanently or at least for a prolonged period.

**Fig. 2:**
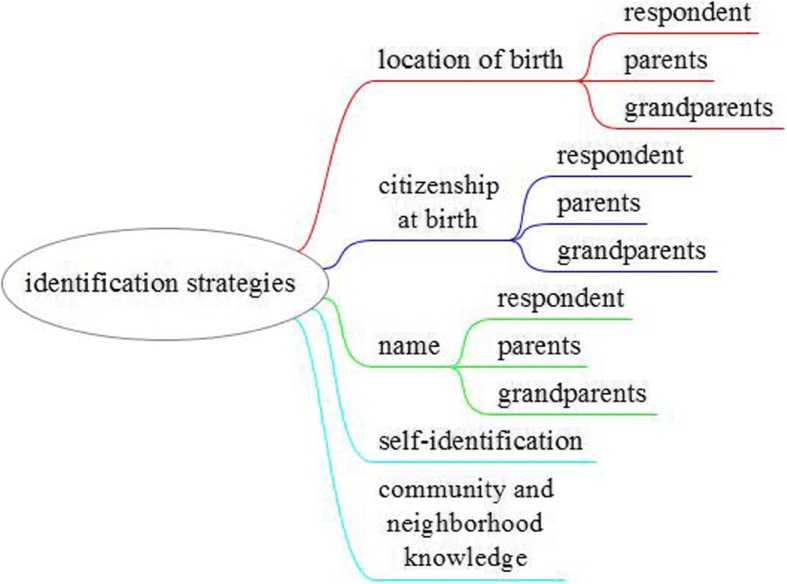
Identification strategies

The experts considered various strategies to identify the aforementioned target population, summarized in Fig. 2. The main identifying characteristic is whether individuals belonging to this population have been born outside the country (first-generation) or descend from someone who was born outside the country of current residence (second and following generations). A less satisfactory identifying characteristic is citizenship, because immigrants may give up their citizenship when they acquire the citizenship of their destination country. Many countries accept dual or even multiple citizenships, therefore citizenship is only useful for sampling if the original citizenship is recorded along with the acquired citizenship(s). If neither place of birth or citizenship is available, other identification strategies can be used, for example typical names of the particular immigrant group, survey questions which ask respondents to identify themselves as members of a particular immigrant group (e.g., as used in the UK census), or knowledge of local communities and neighborhoods (e.g., in snowball sampling techniques).

**Fig. 3:**
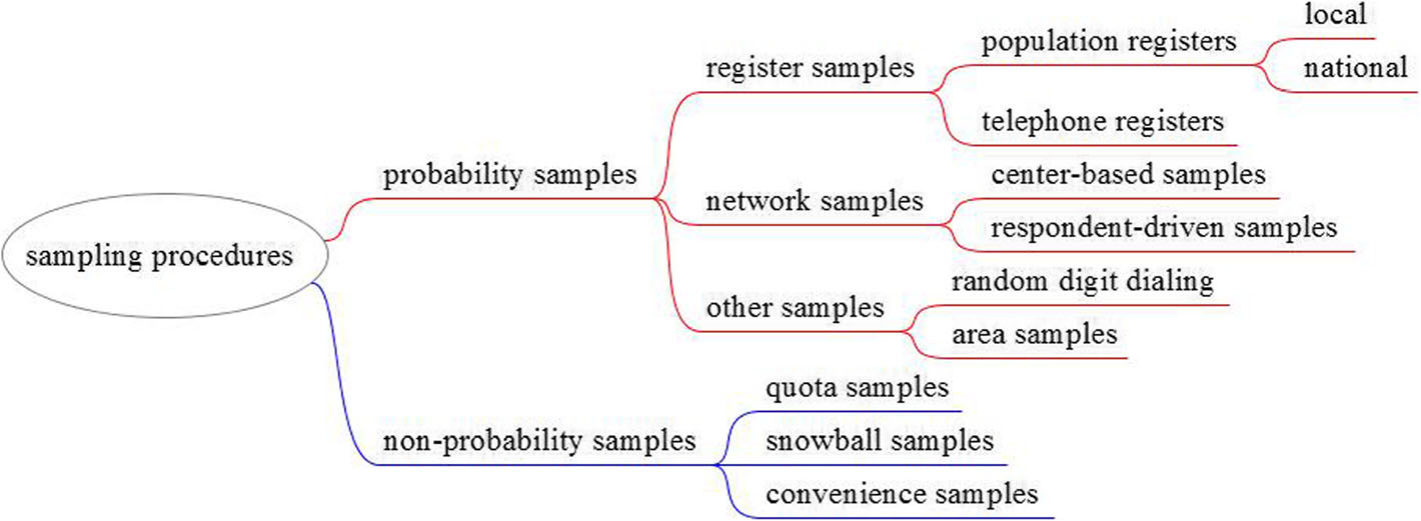
Sampling procedures

Having defined the target population and possible identification strategies, the next question our experts dealt with was how to sample from that population. Several sampling strategies used in migration research have been reviewed (see Fig. 3 for a simplified and non-exhaustive overview of sampling procedures).^4^ There is widespread agreement that for scholarly research, probability samples are the preferred choice because they allow statistical inferences about the (larger) target population.^5^ Ideally, these probability samples should be selected from up-to-date population registers that include *all* members of the target population.

## The countries of our comparison

Given the frame for discussion and comparison developed in “A frame of comparison: definitions, identification strategies, and sampling procedures” section, we ask which of these different options of defining, identifying, and sampling immigrant minorities are feasible across different European countries. Our comparison is based on six migrant destination countries from the European Union: Denmark, Germany, Italy, The Netherlands, Spain, and Sweden. It is an availability sample based on those countries being represented at the conference. It is certainly not a census of all EU member states and important European destination countries are missing (for example the United Kingdom and France; for sampling immigrant minorities in these two countries see Lynn, Nandi, Parutis, & Platt, 2018; Platt, Luthra, & Frere-Smith, 2015 and Simon, 2008). We argue, however, that the group of countries included in this overview provides enough regional, historical, political, and statistical variance to study the problems and pitfalls of cross-national comparative sampling designs for immigrant minorities. A comprehensive comparison of all EU member states certainly needs more resources and is left for future research.

The six countries included in this study span a broad geographical area: Northern Europe (Sweden, Denmark), Continental Europe (Germany, The Netherlands), and Southern Europe (Spain, Italy). They also have different experiences with immigration. After WWII, Sweden, Denmark, Germany, and The Netherlands have primarily been immigration (and not emigration) countries, while Italy and Spain have a longer history of emigration and only recently faced large waves of immigration. Altogether, they represent a significant part of the EU population (44% in 2017) and the immigrants living in EU member states (51% in 2016).^6^

The “old” countries of immigration have had longer time to recognize the limitations of their statistical sources to properly measure the size and understand the (changing) characteristics of their immigrant populations, and to adapt them accordingly. The “new” countries of immigration, Italy and Spain, first have to catch up with this advance in statistical infrastructure. Moreover, both are situated at the external border of the EU and have large informal economic sectors, resulting in high rates of unregistered immigrants, which constitute an additional challenge for the statistical recording of immigrant minorities.

The Migrant Integration Policy Index, which captures the formal institutional arrangements affecting immigrants’ integration, shows that, in 2014, Italy (index value 58), Denmark (59), The Netherlands (61), Spain (61), and Germany (63) have relatively close index positions, above the EU28 average value of 52, while Sweden stands out with an index value of 80 (see the Migrant Integration Policy Index (MIPEX) at http://www.mipex.eu).

**Fig. 4:**
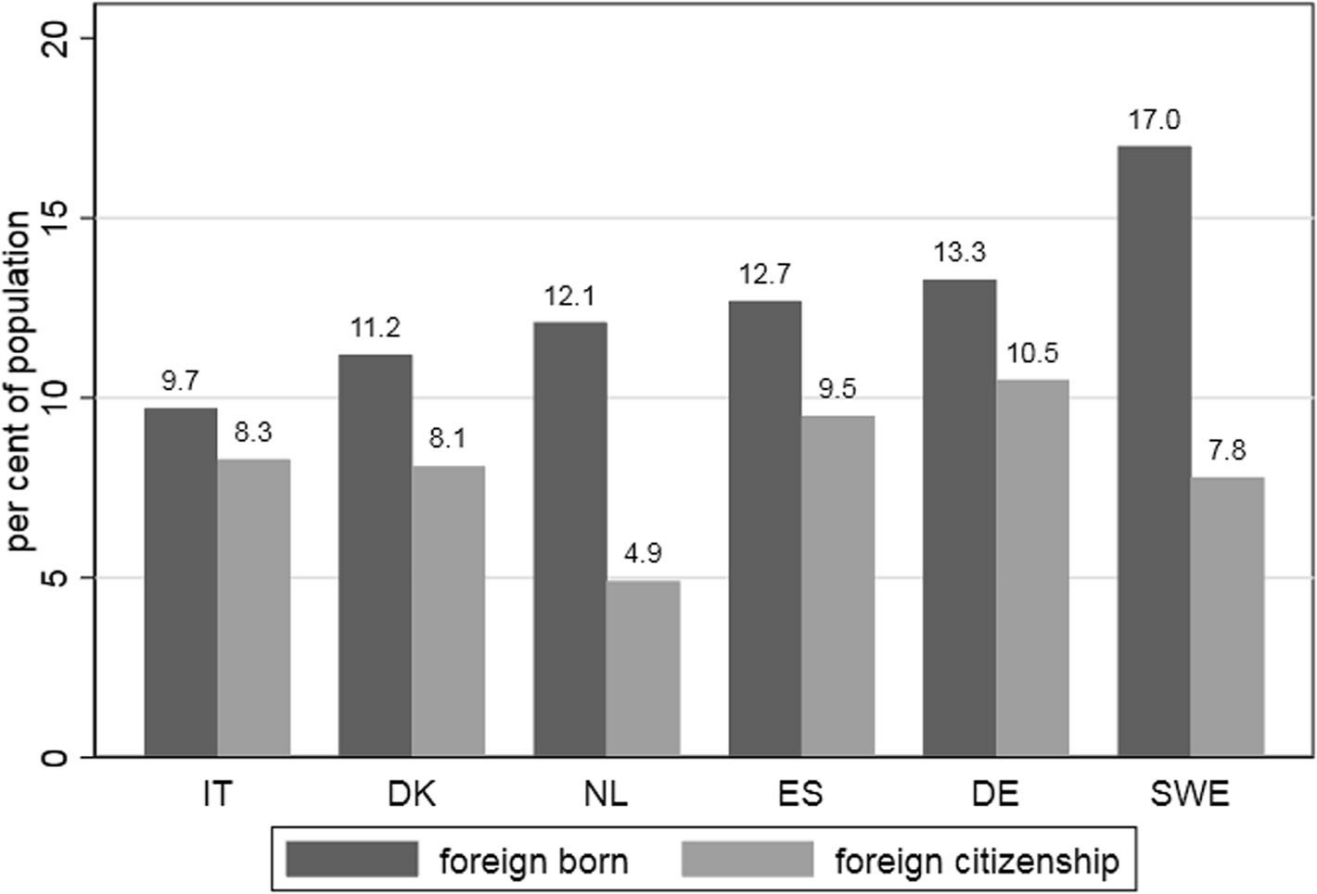
Proportion of immigrants (1 January 2016, per cent of the population). Eurostat (online data code: migr_pop3ctb)

According to Eurostat statistics, all six countries have a sizeable immigrant population. As already mentioned, Eurostat reports only first-generation immigrants with minimum one year of residence in the destination country. Depending on whether country of birth (COB) or citizenship (CS) is used to identify immigrants, the proportion ranged between 9.7 and 17% (COB) or between 4.9 and 10.5% (CS) (see Fig. 4). Not surprisingly, numbers based on citizenship are lower, because some foreign-born nationals naturalize. The discrepancy is largest in Sweden and The Netherlands and has to do with the liberal naturalization legislation in these two countries (until 2000), and, in the Dutch case, additionally to the colonial legacy.

**Fig. 5:**
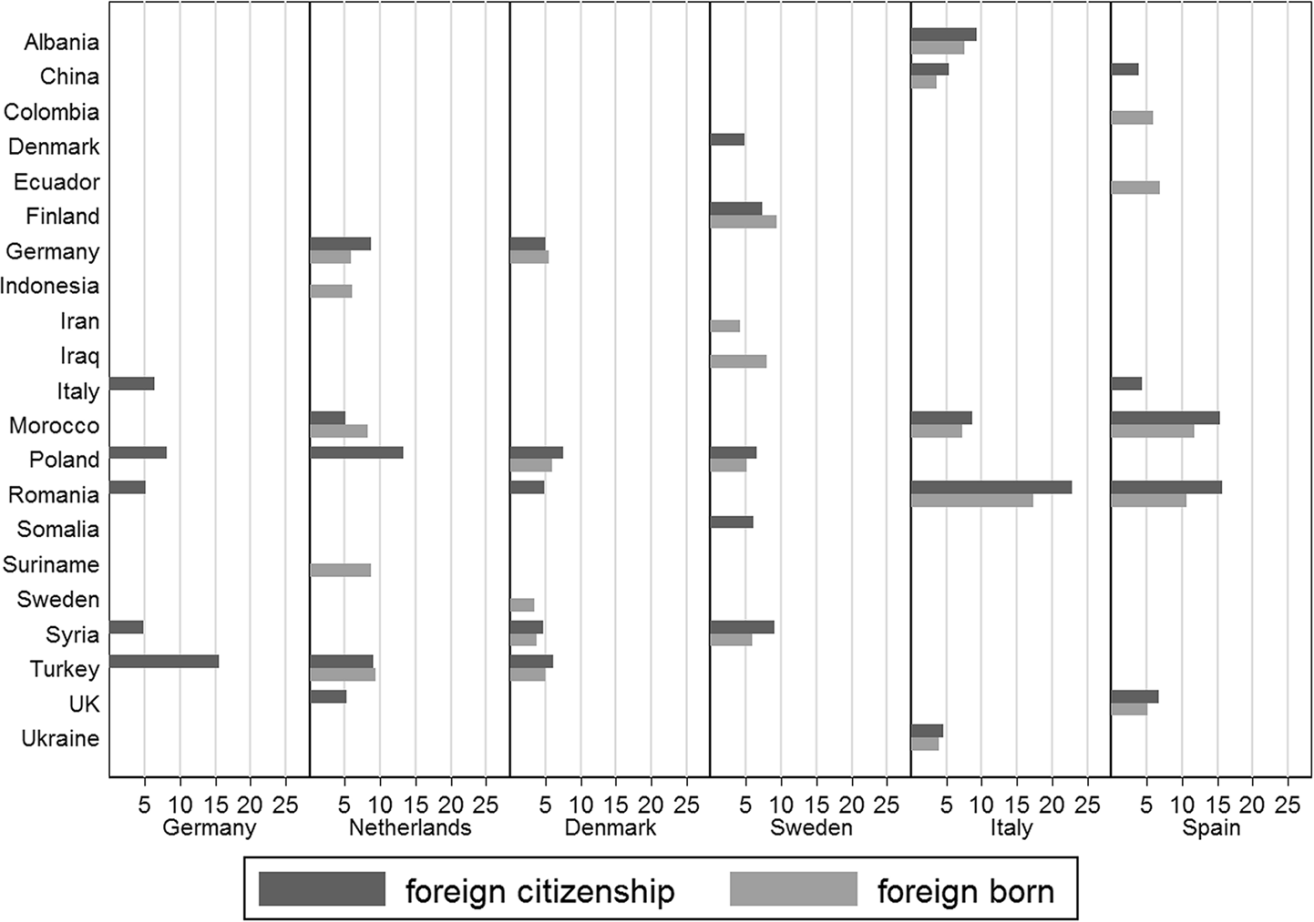
Main countries of citizenship & birth of the foreign/foreign-born population (1 January 2016, per cent). *Notes*: The graph shows for each country of our country sample the five largest immigrant groups as a percentage of the total foreign population in the country (black bars) and the five largest immigrant groups as a percentage of the foreign-born population in the country (grey bars; except for Germany). Eurostat (online data codes: migr_pop1ctz and migr_pop3ctb)

Although quite similar in size, the origins of the immigrant population are quite diverse (see Fig. 5). For example, Polish immigrants are almost everywhere, but not so much in the southern European countries Italy and Spain. Romanians, on the other hand, form sizeable portions of the immigrant population in Italy and Spain as well as in Germany and Denmark, but are not among the top origin countries in Sweden and The Netherlands. Large Turkish communities exist in Germany, Denmark and The Netherlands, but not in the other three countries. Somali, Syrian, and Iraqi immigrants have mostly left their countries due to war, political oppression, and failed governments, but made their way mostly to the Nordic countries Sweden and Denmark (and recently Germany). This list of examples of diverse immigrant populations is easily extended. By and large, the differences can be explained by geographical and/or language proximity between origin and destination countries as well as by historical legacies of colonial empires, the development of labor demand, and immigration and asylum policies in the respective destination countries (for more details see the extensive country descriptions in this Special Issue).

Eurostat data, albeit useful in many ways, is not useful when contemplating sampling immigrant minorities in European countries as it does not depict a full picture: The data includes only first-generation immigrants with minimum one year of residence, and lacks those nuances which are present in the domestic statistical accounting definitions (for an overview on these national accounting practices see Fassmann, Reeger, & Sievers, 2009). Therefore, sampling immigrants (and their descendants) in these countries should be based on national and not on EU-wide harmonized data. But can this be done in a comparative way for the six countries of our study? And how?

## What is possible in Denmark, Germany, Italy, The Netherlands, Spain, and Sweden?

In the following discussion, based on the information included in the country-specific analyses in this Special Issue (Careja & Bevelander, 2018; Salentin & Schmeets, 2017; Serrano Sanguilinda et al., 2017), we have extracted few basic dimensions which allow us to build a broad picture of the sampling frames available. The analyses contain much more information and details relevant for researchers interested in survey-based studies in these countries. Therefore, we strongly recommend scholars to read the articles. The aim of this concluding article is to identify possibilities to sample immigrant minorities in a way which would support comparative research. As probability samples are regarded the cornerstone of sound scholarly survey research, our discussion about the sampling strategies leading to comparable high-quality samples of immigrant minorities will focus on the possibilities to obtain probability samples in the six countries.

From the framework discussed in “A frame of comparison: definitions, identification strategies, and sampling procedures” section, we have derived four larger questions used to organize the available information: (i) What are basic concepts and terminological definitions in the national discourse on migration? (ii) How can the target population of immigrant minorities be identified? (iii) Given the importance of probability samples, do population registers exist that identify the target population and allow scholars to develop a sampling frame? (iv) If such register information is not available, incomplete, unreliable, or not accessible for scientific research, what other sampling designs have been used? In the following section we summarize the main conclusions from the country analyses. Additional information can be found in Table 1, where the six countries are arranged from the left to the right, with countries in which the ideal case of a register-based probability sample is feasible to the left (Denmark, Sweden, The Netherlands, Spain), and countries in which the ideal case is difficult to obtain (Germany) or impossible (Italy) to the right.

### Basic concepts and definitions

*Foreign citizenship* is the identification criterion present in all six countries’ official statistics and government publications. But depending on naturalization rates and citizenship law, this criterion does not allow the identification of all persons with immigrant background. Naturalization of first-generation immigrants depends on residence status, residence duration, language proficiency, etc. But if it comes to children of those immigrants, it becomes quickly quite complicated. For example, while Germany traditionally applied *ius sanguinis* for new-born children, it introduced *ius soli* for the second generation of immigrants in 2000, which grants them two citizenships until adult age and individuals have to choose one of the two until age 23.^7^ In Denmark, to mention another example, it depends on place of birth and citizenship of the parents, with the mother’s citizenship being somewhat more important. It should also be noted that registers may record only one citizenship if an individual has several, and may not record their former citizenship if the individual acquires the host country’s citizenship. Moreover, it is important to acknowledge that there are individuals entering the country who already have acquired the country’s citizenship or who acquire it more or less automatically, because they themselves or their ancestors emigrated out of the country in former years. Important examples in this respect are German (late) emigrants (“*(Spät)Aussiedler*”)) from the former eastern European countries, which after the fall of the Iron Curtain migrated to Germany; or the children of Jewish emigrants from Germany due to the Holocaust, for whom it has become attractive to own a second (“European”) citizenship in addition to their first one (Harpaz, 2013, 2015). While the latter do not necessarily immigrate into Germany and rather use the German passport for easier travelling, the former certainly do.

*Country of birth* provides a much more comprehensive account of the immigrant population, as the numbers in Fig. 4 suggest. However, with respect to official statistics and government publications, *foreign born* is only used in The Netherlands, Sweden, and Spain. Denmark distinguishes between persons of *Danish origin*, *immigrants*, and *descendants* of immigrants, with immigrants identified by country of birth. Germany uses the term *migration background* to identify foreign born individuals and their children, but this information is based on samples (the German “Mikrozensus”) and does not come from registers or census data. Finally, foreign born is not a term in Italian official statistics and government publications, which only acknowledge *foreign citizen*.

The Netherlands and Denmark also differentiate their information with respect to first and second generations of immigrants, i.e., between immigrants and their descendants. In principle, a similar distinction can also be made in Germany, which differentiates individuals with migration background whether they have or have not own migration experiences. The later ones are those who have been born in Germany (and hence, not migrated to Germany). Nevertheless, this group does not comprise the whole second generation, because there are children of immigrants that have been born outside Germany. In Sweden, identification of children of immigrants based on official statistics is more difficult, as the Swedish-born category also includes children of Swedish ethnic origin.

This brief overview underlines that any comparative sampling endeavor must take into account the fact that the officially used terms are not perfectly overlapping, and therefore a careful preliminary mapping of the population of interest as captured by country-specific terminology must be undertaken.

### Available registers and coverage

As Table 1 in the Appendix shows, all six countries have population registers, either at the national or the local (municipal) level which, in principle, include the aforementioned immigrant groups as long as they hold a valid residence permit. Local registers exist in Germany (“Melderegister”) and in Italy (“Anagrafe”), while registers in The Netherlands (BRP – Basic Population Register), Denmark (“Det Centrale Person Register”), Sweden (“Folkbokförd”), and in Spain (“Padrón”) are maintained at the national level, even though the data may come from local authorities. As will be discussed below, identification problems are smallest for first-generation immigrants, because many of them can be identified by their foreign citizenship or country of birth, while second generation immigrants, foreign-born citizens, refugees, or asylum seekers are more difficult to identify either because they do not yet have a residence permit or because the citizenship criterion does not work with foreign-born citizens and naturalized second generation immigrants. Refugees and asylum seekers are kept in separate registers in some countries, and hence may be identifiable, for example, in The Netherlands, Denmark and Sweden, but not in the other countries.

Needless to say, sampling frames are much more easily derived from centralized registers. If registers are available only at the local level, one has to draw a sample of localities and then, from those localities, draw samples from local registers.^8^ These multi-stage cluster samples are (in statistical terms) less efficient than simple random samples and moreover, need more resources for their implementation.

After arrival (usually within few weeks or months) and given a minimum duration of residence, registration is compulsory in all countries except in Italy. However, incentives for registration vary significantly between countries and possibly are enforced differently. For example, in Germany, fines can be imposed for infringement, but is rarely enforced due to government authorities’ lack of resources to control unregistered residents. In the other countries, access to welfare benefits and services is contingent on being a legal resident, and non-registration may have negative consequences. For example, in Sweden and Denmark each legal (i.e. registered) resident has their own personal identification number, which makes opening a bank account and accessing daily public services easier. In Spain, registration is mandatory for accessing basic services, such as primary health care and education. There are also public campaigns for registration because municipal budgets depend on the number of registered residents. However, statistical information on the amount of undercoverage is missing in almost all countries, and it is difficult to estimate. Given the voluntary inscription rules in Italy, it is reasonable to expect that immigrants are strongly underrepresented in Italian population registers compared to registers in the other countries.

Compared to the risk of undercoverage, overcoverage seems to be the larger problem because there are hardly any incentives for deregistration if individuals leave their place of residence and this risk is especially high for mobile persons such as immigrants. Countries implement various strategies to “clean” their records, with varying rates of success. Table 1 in the Appendix shows some scattered evidence of the amount of overcoverage for some countries. In principle, this information should also be available from the last census round in the EU, but systematic research is missing here.

The discrepancy between the out-movement of immigrants and the register information at a given point in time can be problematic for obtaining a representative sample of immigrants. These problems are likely to increase if out-migration is not random, which is a reasonable expectation. In the EU context, immigrants of EU origin can more easily move across borders, and this increased mobility means that they are more likely than other groups to have left the country but failed to remove themselves from the register.

### Content of the registers

Parallel to the issues of under- and overcoverage, the content of the registers is of crucial importance. Are all the necessary variables (see Fig. 2) included in order to identify different groups of migrants, to stratify sampling units according to important socio-demographic characteristics (say, age and gender), or to apply alternative sampling strategies, such as identification strategies based on names? Moreover, how complete and reliable is this information? And finally, is all this information accessible to scholars from academia?^9^

For each of the six countries, the authors of the country-specific articles rated the available variables with respect to their completeness and reliability (see Table 1 in the Appendix). As previously discussed, it is not surprising that the citizenship criterion is available in all countries, has a low amount of missing data, and the existing information is highly reliable. The same is true for possible stratifiers such as age and gender. However, already when it comes to additional citizenships in order to (at least partly) identify naturalized immigrants, registers in five of the six countries do not include this information. Country of birth is available with similar quality (few missings; existing information highly reliable) in all countries, except in Italy. In Germany, however, the amount of missing information related to country of birth is higher than for citizenship and in some municipalities country of birth is withheld by the local authorities. Hence, identifying foreign-born individuals is the least problematic only in The Netherlands, Denmark, Sweden, and Spain. The same is true if one wants to differentiate immigrants according to their time of residence in the host country. Date of arrival is not available in Germany and Italy, only for the other four countries (in Spain a special petition is needed to access this information). In order to identify second generation immigrants, information on their parents is needed. These links are only available in the countries with a long tradition of register-based statistical accounting (The Netherlands, Denmark, and Sweden) and for minors with a migration background in Germany, but not in Italy and Spain. Finally, identification of immigrants by names is problematic in of itself. Inherent problems aside, name identification is also difficult to apply in a comparative sampling design because of data-protection regulations in some of the six countries.

### Accessibility and statistical infrastructure

Table 1 in the Appendix also gives information on the accessibility of the registers for scholars from academia and on the national statistical infrastructure that researchers can use to design their research, such as regular national reports or regular data sources (censuses, large-scale surveys) providing national statistics on migration and other relevant side information for sampling designs. Registers are available for scientific research in all countries except Italy, however, the countries differ widely in the conditions attached to access. For example, the research has to be in the public interest (Germany), supported by the public administration (Spain), or done in cooperation with a national (domestic) research institute or university (The Netherlands, Denmark, Sweden). Certainly, these conditions are difficult to fulfill for foreign researchers and hence, cooperation or affiliation with national researchers is always useful, if not necessary. Linkage with other registers is also possible (and available for researchers) in the countries with a long tradition of register-based statistical accounting (such as Denmark or Sweden), but not in Germany with its historical reluctance towards centralized and linked registers after the experience of the Third Reich. In Italy, only the national statistical office ISTAT has the possibility to link information in registers.

### Other registers and sampling methods

Finally, Table 1 in the Appendix mentions some other registers and sampling methods that have been or could be used in the six countries to sample immigrants. Electoral, foreigner, or telephone registers exist, but are not accessible for scientific research (e.g., the foreigner register in Germany); have severe coverage problems (telephone registers in all countries), or are not a viable alternative because they are based on the population registers (electoral registers). Screening the whole population for immigrants by area-based sampling methods or random dialing of telephone numbers is feasible in principle, but in practice is connected with excessive search costs because immigrants are still a minority. Costs can be reduced in area-based sampling methods due to the fact that immigrants tend to concentrate in particular geographical units. However, using the geographically clustered nature of the target population increases the risk of overlooking immigrants outside the clusters and decreases the efficiency of estimates based on these samples. However, this cost-reducing method cannot be applied to samples based on other than geographical units, such as blocks of telephone numbers, and even more importantly, due to prepaid and foreign mobile phones, the total population of telephone numbers is often unknown. If all these registers and sampling methods fail, respondent-driven or centre-based sampling techniques are the only practical alternatives.

## Discussion and conclusion

The main conclusion we draw from this comparison is that, even with only six countries, an identical register-based sampling design is difficult. Although all the surveyed countries have population registers (in one form or another), the likelihood of obtaining probability samples of immigrant minorities exists only in Denmark, Sweden, The Netherlands, and Spain (hereafter the “register” countries), where population registers allow the extraction of country-wide samples. In Germany, the ideal case is difficult to achieve because registers are decentralized at the level of municipalities (which can deny access) and have to be combined by the survey researchers. Finally, in Italy, registers are also administered by the municipalities, and are not accessible for scientific research.

However, the sheer availability of registers does not guarantee that they provide enough information to identify the target population. Again, the situation in the four “register” countries is more favorable because they also include information about nationality and country of birth, for the parents of the registered individuals (with the exception of Spain), so that one can identify second generation immigrants. In principle, nationality and country of birth are also available in Germany, but data quality on country of birth is low and hence, it is no surprise that official German statistics only publish data on foreigners (see Fig. 5, in which the category “foreign born” is completely missing for Germany). In addition to the problem of identifying the target population comes the question of whether registers cover all members of the target population. All registers in our survey are affected by over- and undercoverage. Overcoverage seems to be the larger problem because the national regulations hardly provide any incentives for deregistration, which of course has negative effects on the correct registration of mobile persons such as migrants. Undercoverage is not negligible, but less of a problem because quite strong incentives exist to register in most countries. Unfortunately, the exact amount of over- and undercoverage is unknown in many countries and is likely to vary with the group of immigrants: immigrants who can move easily across borders, such as EU citizens, are more likely to be affected by overcoverage than their less mobile counterparts. Finally, from the practical point of view for *comparative* survey research, all these methodological challenges remain academic discussions if registers are not accessible for foreign scholars. Certainly, accessibility has to be negotiated, but it seems that access is only possible in cooperative networks including scholars and research institutes from each country.

Hence, the main conclusion from our comparison is that the ideal case of nation-wide register-based probability samples is only feasible for a very small group of European countries. Depending on the aim of research, this can be problematic: for example, if researchers are interested to study the effect of contexts of reception on integration outcomes, more contextual variation, i.e. more countries, is needed. However, enlarging the country sample almost certainly^10^ necessitates the use of alternative sampling strategies for certain countries and a mixture of different sampling procedures across countries raises questions about the comparability of the resulting samples and yet, it currently seems to be the only feasible option for nation-wide samples. In this situation, we argue that more research on these mixed-procedure sampling designs is required, in order to understand the extent to which the resulting samples are comparable.

Focusing on regional units, such as cities in different countries, seems to us a practical alternative. Several migrant survey projects have already taken this direction: Multicultural Democracy and Immigrants’ Social Capital in Europe (LOCALMULTIDEM). The Integration of the European Second Generation (TIES); and the Socio-Economic Inclusion of Migrant EU Workers in 4 Cities.^11^ These projects include cities in different countries and implement a variety of sampling procedures, given the availability of local sampling frames, or lack thereof. We argue here for a continuation in this direction, plus for future endeavors to use the same (or as similar as possible) sampling frames to ensure the comparability of the final samples. There are, in our opinion, several important reasons for which sampling should be focused at city level. First, the context of reception is not only determined by national characteristics, because many integration policies are developed on the local level. Therefore, a multi-level design (cities within countries) would be a much better representation of the different contexts of reception. Secondly, immigrants are not randomly distributed across the host country. They often concentrate in urban areas (with the important exception of immigrant workers in agriculture). Thirdly, such a design is also feasible for projects with more limited budgets. Fourthly, as our concern is on ensuring that sampling starts from similar sampling frames, focusing on cities has the advantage in that, on the one hand, it opens the possibility to include countries where only local population registers exist (e.g., Germany), and on the other researchers can implement network-based sampling as an alternative to register-based sampling. All network sampling procedures work best in areas where immigrants concentrate. Network sampling techniques, such as centre-based or respondent-driven sampling (see Fig. 3), have been successfully applied in Italy (see the article on Italy and Spain in this Special Issue) and in other countries (Arnholtz & Hansen, 2013; Crul, Schneider, & Lelie, 2012; Reichel & Morales, 2017). And finally, local areas are likely to be the best laboratory to test and improve the coverage and selectivity problems of our sampling frames. For example, in the case of network sampling procedures, it has been found that they over-represent individuals well integrated in immigrant communities and visible in centers of immigrant aggregation (McKenzie & Mistiaen, 2009; Reichel & Morales, 2017). We recognize that this solution might not satisfy all research interests, but, in our opinion, it may set some research projects on a solid footing when it comes to discussing the quality of the samples.

## Appendix

**Table 1:** Summary of the expert reviews

Item	Denmark	Sweden	The Netherlands	Spain	Germany	Italy
1. Terminology in official statistics and government publications						
Terms	person of Danish origin, immigrant, descendant	foreign born, foreign citizen, foreign background, asylum seekers	person with migration background; non-dutch nationals; foreign born; first and second generation	foreign born, foreign citizen, immigrant (incl. Spanish citizens)	foreigner, person with migration background, asylum seeker, Aussiedler, refugee, minority	foreign citizen
2. Availability and access to population registers						
Name of the register	Population register (Det Centrale Person Register)	Population register (Folkbokförd)	Basic Population Register (BRP)	Municipal population register (Padrón)	Municipal population register (Melderegister)	Municipal population register (Anagrafe)
Responsible institution	Statistics Denmark	Statistics Sweden	Statistics Netherlands	Statistics Spain (INE)	Municipality	Municipality
Access for scientific research	Affiliation with a Danish research institute necessary	Affiliation with a Swedish research institute necessary	Dutch Universities and Research Institutes	Official support from some unit of the Spanish Public Administration (excl. universities)	possible if in the public interest	no
Access for foreign researchers	possible dependent on additional conditions	possible dependent on additional conditions	possible dependent on additional conditions	Official support from some unit of the Spanish Public Administration	possible with restrictions if in the public interest	no
Linkage with other registers	possible	possible	possible	possible - specific request and INE’s assessment	impossible	possible only by ISTAT
3. Immigrant identification						
Nationality	yes	yes	yes	yes	yes	yes
Country of birth	yes	yes	yes	yes	missings	no
Date of arrival	yes	yes	yes	Date of 1st registration, accessibility restricted	no	no
Parent information	yes	yes	yes	no	only for minors	no
Personal name	yes	personal number	no	yes	yes	no
Age	yes	yes	yes	yes	yes	yes
Gender	yes	yes	yes	yes	yes	yes
4. Registration						
Legal regulations	compulsory for all individuals residing 3 or 6 months in DK	compulsory for all individuals residing 1 year in SWE	compulsory for all individuals residing at least 4 months in NL	compulsory for municipalities to register all residents (incl. irregular immigrants)	compulsory for all individuals after 2 weeks from moving into the respective municipality	voluntary inscription for individuals with valid permits of residence
Incentives for registration	access to welfare benefits and services, open bank accounts, access to daily services	access to welfare benefits and services, open bank accounts, access to daily services	access to welfare benefits and services	access to public health care and other public services such as primary schools, proof of residence for regularisation, campaigns by municipalities	fines can be imposed for infringement	access to welfare benefits and services
Estimate of undercoverage	33,000 in 2013 [1]	10.000–50.000 (2.000–3.000 children)	1% of population [2] 9% of first generation		3% of all foreigners in 2011 [4]	7% of immigrant population [3]
5. Deregistration						
Legal regulations	compulsory for all individuals staying abroad > 6 months		compulsory for all individuals staying abroad at least 8 months	compulsory, but without sanctions	compulsory for all individuals leaving DE	voluntary
Incentives for deregistration	none		no real incentives	none, but extinction procedure (since 2007) and periodical residence check (since 2013)	none	none
Estimate of overcoverage	7500 individuals 0.14% of the population [5]	4–8% of total foreign-born population	0,2% of total population [2]	250,000 non-EU in 2007 400,000 (87% EU) in 2013	11.3% of all foreigners in 2011 [4]	
6. Alternative sampling frames (existence, accessibility, and problems)						
Electoral register	yes, based on population register, problematic	yes, based on population register, access only when applying to regional authorities	yes, problematic	yes, no access	yes, based on population register, no access	no
Foreigner register	yes, largely overlaps with population register	yes, problematic	yes, problematic	yes, no access	yes, no access	no
Telephone register	yes, problematic	yes, problematic	yes, problematic	yes, no access	yes, problematic	yes, unreliable
Area-based sampling	yes, no evidence	yes, problematic	yes, problematic	yes, problematic	yes, problematic	
Random dialing	yes, problematic	yes, problematic	yes, problematic		yes, problematic	
Respondent driven	possible, few studies			yes, problematic	possible, no evidence	
Center-based sampling	possible, no evidence				possible, no evidence	yes
7. Data infrastructure						
Reference statistics	population register	Population register (Folkbokförd)	Basic Population Register (BRP) (gets updated every month)	Census 2011; Padrón 1st January 2017	Microcensus (annual 1% survey of the population), Census 2011 (regional breakdown only to Landkreis)	Census 2011
8. Important national reports						

*Denmark*: Danmarks Statistik (2016): Indvandrere i Danmark 2016 [Immigrants in Denmark 2016]. København. Retrieved from http://www.dst.dk/pubfile/20704/indv2016. *Sweden*: Statistics Sweden (2016): Retrieved from http://www.scb.se/Statistik/_Publikationer/UF0529_BR_2015A01_A40BR1603.pdf; http://www.scb.se/contentassets/dc0958cc6c9f4747aa2c4a699472b2c9/le0105_2016a01_br_be57br1601.pdf. *The Netherlands*: Ooijevaar, J. & Bloemendal, C. (Eds.) (2016): Jaarrapport Integratie 2016 [Yearly Integration Report 2016]. Den Haag/Heerlen/Bonaire: CBS. *Spain*: Informe Encuesta Nacional de Inmigrantes (ENI – 2007) Retrieved from http://www.ine.es/daco/daco42/inmigrantes/informe/eni07_informe.pdf; Documentos del Observatorio Permanente de la Inmigración (2004–2015) Retrieved from http://extranjeros.empleo.gob.es/es/ObservatorioPermanenteInmigracion/index.html. *Germany*: Die Beauftragte der Bundesregierung für Migration, Flüchtlinge und Integration (2012): “Zweiter Integrationsindikatorenbericht.” [Second Report on Integration Indicators]. Berlin: Die Beauftragte der Bundesregierung für Migration, Flüchtlinge und Integration; Die Beauftragte der Bundesregierung für Migration, Flüchtlinge und Integration (2014): “10. Bericht der Beauftragten der Bundesregierung für Migration, Flüchtlinge und Integration über die Lage der Ausländerinnen und Ausländer in Deutschland.”: [Tenth Report of the Federal Government's Commissioner of Migration, Refugees and Integration about the Living Situation of Foreigners in Germany]. Berlin: Die Beauftragte der Bundesregierung für Migration, Flüchtlinge und Integration. *Italy*: Cesareo V. (ed.) (2017): The Twenty-second Italian report on Migrations 2016. Milano: Mc Graw-Hill Education

*Notes*: [1] Retrieved from http://www.rockwoolfonden.dk/en/articles/marked-increase-in-recent-years-in-the-number-of-illegal-immigrants.

[2] Retrieved from https://www.cbs.nl/nl-nl/achtergrond/2017/09/haalbaarheidsstudie-gewoonlijk-verblijvende-bevolking.

[3] Blangiardo, G.C. (2016): Statistical aspects. In Cesareo V. (Ed.). (2017). *The Twenty-second italian report on Migrations 2016*. Milano: Mc Graw-Hill Education.

[4] Bechtold, S. (2013). Analyse der durch den Zensus verursachten Abweichungen bei den Einwohnerzahlen. Vortrag im Rahmen der Statistischen Woche, Berlin, 18.9.2013 [Analysis of deviating population numbers in the Census. Presentation at the "Statische Woche", Berlin, 18.9.2013]. Retrieved from https://www.zensus2011.de/SharedDocs/Downloads/DE/Praesentationen/2013_09_Statistische_Woche_Vortrag_Dr_Bechtold.pdf?__blob=publicationFile&v=6.

[5] Statistics Denmark. Retrieved from www.dst.dk/en/Statistik/dokumentation/documentationofstatistics/immigrants-and-descendants/accuracy-and-reliability

## Footnotes

1 LISS also included a separate (‘non-western’) immigrant panel study (apart from the regular LISS panel) from October 2010 up until December 2014.

2 The German Data Archive has published a collection of migrant surveys for secondary analysis at: Retrieved March 31, 2018, from http://www.gesis.org/home/angebot/daten-analysieren/weitere-sekundaerdaten/uebersichten/migration-research/.

3 The offspring are not necessarily born in the country. The essential point is here that they live in the country and have a legal status either on their own or derived from their parents.

4 Figure 3 does not include various complex sampling designs such as cluster, stratified, or multi-stage sampling.

5 Figure 3 includes center-based or respondent-driven sampling within the probabilistic group, but some researchers prefer to consider them semi-probabilistic procedures.

6 Own calculations based on Eurostat population and immigration figures, online data codes: tps00001, migr_imm1ctz.

7 The compulsory choice (“Optionspflicht”) has been abolished in 2014 (Bundesministerium des Innern, n.d.; Bundesregierung, 2014).

8 Remarkably, the IAB-SOEP Migration Sample did not use the local population registers. The sample was drawn from the Integrated Employment Biographies (IEB), a database comprising all employees, unemployed persons, and participants in government initiatives to promote employment in Germany. IEB is maintained by the Institute for Employment Research (IAB) of the Federal Employment Agency.

9 Scientific research produced in or on behalf of government institutions often has access to these scarce resources. For example, in Germany, the Institute for Employment Research (IAB), the research center of the Federal Office for Migration and Refugees (BAMF-FZ), and the German Socio-Economic Panel (SOEP) have forged a partnership to establish a household panel study on recent refugee migration to Germany: The IAB-BAMF-SOEP Refugee Survey. For this project, they were able to use the Central Register of Foreigners held by the German Federal Office for Migration and Refugees (BAMF) as a sampling frame.

10 Exceptions are, of course, subgroups of immigrant minorities that are accessible through other registers. For example, CILS4EU is focusing on the children of immigrants, which can be accessed through the schools they attend (Retrieved from the CILS4EU website http://www.cils4.eu).

11 For the third project: retrieved March 31, 2018, from http://ec.europa.eu/social/BlobServlet?docId=14423&langId=en.
